# Mechanical instability of the lumbar spine following intervertebral disc injury: a comparison of two injury methods

**DOI:** 10.1080/23335432.2025.2581469

**Published:** 2025-10-30

**Authors:** Fangxin Xiao, Wendy Noort, Juliette Lévénez, Jia Han, Jaap H. van Dieën, Huub Maas

**Affiliations:** a Department of Human Movement Sciences, Faculty of Behavioural and Movement Sciences, Vrije Universiteit Amsterdam, Amsterdam Movement Sciences, Amsterdam, The Netherlands; b School of Exercise and Health, Shanghai University of Sport, Shanghai, China; c College of Rehabilitation Sciences, Shanghai University of Medicine and Health Sciences, Shanghai, China; d Faculty of Health, Arts and Design, Swinburne University of Technology, Hawthorn, VIC, Australia

**Keywords:** Low back pain, intervertebral disc injury, animal model, spine instability, spine mechanics, needle puncture

## Abstract

Intervertebral disc (IVD) degeneration is a potential contributor to low-back pain. While experimental IVD injury models have demonstrated IVD structural changes, the early mechanical consequences remain unclear. This study aims to assess and compare the effects of two IVD injury models on lumbar spine instability and assess back musculature adaptations to IVD injury. Thirty-one adult male Wistar rats were assigned to three groups: IVD knife stab lesion (knife), IVD needle puncture (needle), and sham surgery control (control). In the knife and needle groups, L4/L5 IVDs were injured at 14 weeks of age. One to two weeks post-intervention, lumbar multifidus (MF) and medial longissimus (ML) muscles were excised, L4-L5 spinal segments were harvested for mechanical testing, and IVDs were collected for histology. The needle group exhibited lower peak stiffness, peak moment and hysteresis than controls in flexion, with no difference in lateral bending. IVD height and area did not differ between groups, but the needle group had a smaller nucleus relative to the annulus area compared to controls. Morphological changes were observed in both injury groups. The needle group showed a higher normalized ML mass, while the normalized MF mass was unchanged. In conclusion, lumbar spine instability was successfully established via IVD needle injury in the rat.

## Introduction

Low-back pain (LBP) has been a leading cause of years lived with disability worldwide (Hartvigsen et al. 2018) and spinal instability may be one of the contributing mechanisms (Panjabi 2003). Spinal instability is clinically defined as hypermobility under physiological loads (Farfan and Gracovetsky 1984), which has been observed in people with LBP (Fujiwara et al. 2000a; Passias et al. 2011) and may lead to altered motor control and load sharing among spinal structures (van Dieen et al. 2003, 2019).

IVD degeneration is significantly correlated to instability (Knutsson 1944; Fujiwara et al. 2000a; Fujiwara et al. 2000b; Tanaka et al. 2001; Kong et al. 2009; Galbusera et al. 2014; Swanson and Creighton 2020), shown as increased segmental mobility with increasing severity of IVD degeneration up to Grade IV (Fujiwara et al. 2000a; Tanaka et al. 2001). Experimental IVD disruption has been used to induce IVD degeneration in various animal models (Jin et al. 2018; Desmoulin et al. 2020; Poletto et al. 2023), especially in rodents (Shi et al. 2018). Mechanical injury models, including depressurization of the nucleus pulposus (NP) and/or disruption of the annulus fibrosus (AF), for which scalpel stabbing or needle puncturing are most frequently used (Alini et al. 2008; Jin et al. 2018), allow precise control over the severity and timing of the injury (Alini et al. 2008; Poletto et al. 2023).

Physical disruption of the IVD structure has been demonstrated to induce rapid IVD degeneration. Inflammatory responses (Ulrich et al. 2007), increased catabolic (Chen et al. 2016) and fibrotic activity (Glaeser et al. 2020), and downregulated main NP matrix gene and aggrecan gene expression levels (Glaeser et al. 2020) were detectable 1 week after injury. MRI imaging of rat tail IVD confirmed severe microstructural degeneration, reflected in diffusion properties, 1 week after IVD puncture (Li et al. 2019). Furthermore, morphological changes (Rousseau et al. 2007; Ulrich et al. 2007; Korecki et al. 2008; Chen et al. 2018; Maas et al. 2018; Mosley et al. 2020) reduced NP chondrocytes (Chen et al. 2018) and increased apoptosis (Korecki et al. 2008) were found. Injured discs exhibited decreased height (Kim et al. 2011; Lai et al. 2016), a disorganized AF, decreased NP size (Rousseau et al. 2007; Maas et al. 2018), and blurring of the interface between AF and NP (Poletto et al. 2023). These molecular and morphological changes may affect the IVDs' mechanical properties (Yerramalli et al. 2007; Iatridis et al. 2013; Fontana et al. 2015).

However, our understanding of the mechanical consequences of IVD injury is limited. In rats, in vitro studies have shown that IVD injury acutely compromised bending (Xiao et al. 2023) and torsional (Wang et al. 2022) mechanical properties. In vivo studies showed that, despite the presence of structural degenerative changes, IVD mechanics was not affected by injury neither after 4 (Rousseau et al. 2007) nor after 6 weeks (Mosley et al. 2020).

The mechanical stability of the spine is critical to its proper functioning, for example, to deal with instantaneous changes in spinal posture (Panjabi 1992). Spine stability is maintained by a combination of three subsystems (Panjabi 1992; Stokes and Gardner-Morse 2003): (1) the passive subsystem (vertebrae, intervertebral discs, and ligaments); (2) the active subsystem (muscles and tendons surrounding the spinal column); (3) the neural control system (peripheral and central nervous systems). When there is dysfunction in one of the spinal stabilizing subsystems, adaptation in one or more of the other spinal stabilizing subsystems (e.g. musculotendinous subsystem) may occur (Panjabi 1992; Fujii et al. 2019). For instance, decreased multifidus muscle (MF) mass was observed 1 week after rat lumbar IVD injury, while longissimus muscle (ML) mass tended to increase (Maas et al. 2018). Thus, an understanding of the short-term effects of IVD injury on its mechanical properties, as well as the link between structural and mechanical changes is required to better evaluate the (neuro)mechanical effects of IVD injury.

This study aimed to (1) assess and compare the effects of two IVD injury models on lumbar spine instability and (2) assess adaptations of back musculature to IVD injury. We hypothesized that (1) IVD injury changes the bending mechanics of the spinal segments, including reduced peak stiffness, peak moment, and hysteresis. (2) IVD injury will cause MF atrophy, while the ML mass will increase or remain unchanged.

## Materials and methods

### Animals

A total of 31 adult male Wistar rats (*Rattus norvegicus*) were assigned to three groups: IVD knife stab lesion (knife, *n* = 8), IVD needle puncture (needle, *n* = 14), and sham surgery control (control, *n* = 9). The rats in the knife and control groups were randomly assigned within one study, with sample size determined by a prior power analysis (power = 0.8, alpha = 0.5) using G*Power (Faul et al. 2007), while those in the needle group came from a separate study. Only male rats were used because of the sex difference in IVD’s structural and mechanical responses to injury (Mosley et al. 2019). Surgical and experimental procedures were in agreement with the guidelines and regulations concerning animal welfare and experimentation set forth by Dutch law on animal research in full agreement with the Directive 2010/63/EU, with local approval by and under supervision of the local Animal Welfare Body, and approved by the Central Commission for Animal Experiments of the Netherlands Government at the Vrije Universiteit Amsterdam (Permit Number: AVD11200202115388).

### Surgical procedures for IVD injury

Body mass during surgery and sacrifice are reported in Table 1. IVDs were injured when the rats were 14 weeks old. Carprofen (3 mg/kg, Rimadyl®, Zoetis B.V., Capelle aan den IJssel, The Netherlands) was administered subcutaneously 12 h before surgery. Carprofen and buprenorphine (0.02 mg/kg, Buprecare®, Ecuphar NV, Oostkamp, Belgium) were administrated subcutaneously 30–60 min before surgery. The rats were anaesthetized using isoflurane (induction: 3–5%, maintenance: 1–2%) and placed on a heating pad. A few minutes before skin incision, ropivacaine (2 mg/kg, Fresenius Kabi Norge AS, Halden, Norway) was injected into the skin. All surgeries were performed under aseptic conditions. Body temperature, breathing rate, heart rate and anaesthetic depth were monitored every 10–30 min. Two days post-surgery, carprofen was administered subcutaneously. Three to five days post-surgery, carprofen was provided in drinking water (0.06 mg/ml) and mixed into food. After surgery, the rats were allowed to move freely in their cage with access to food and water *ad libitum* at a 12-h day–night cycle. Body mass changes were monitored for 5 days. The rats were housed in pairs, except for the first 24-h post-surgery, when the two rats in the same cage were separated by a cage divider.

**Table 1.** Body mass, muscle mass and IVD histology.

		Body mass (T0) (gram)	Body mass (T1)^&#^ (gram)	IVD height (mm)	IVD area (mm^2^)	NP area (mm^2^)	Relative NP area^#^	Normalized MF mass	Normalized ML mass
Sample size	Control	9	9	9	9	9	9	8	7
	Knife	8	8	8	8	8	8	8	8
	Needle	14	14	13	9	9	9	14	14
Mean (SD)/median (IQR)	Control	406 (56)	442 (51)	1.38 (0.23)	9.43 (2.02)	1.09 (0.278)	0.128 (0.073)	0.00116 (0.00011)	0.00185 (0.00011)
	Knife	419 (73)	440 (47)	1.27 (0.26)	10.3 (1.87)	1.01 (0.388)	0.089 (0.057)	0.00113 (0.00015)	0.00188 (0.00038)
	Needle	356 (34)	373 (26)	1.28 (0.26)	10.2 (1.48)	0.562 (0.46)	0.048 (0.089)	0.00108 (0.00012)	0.00222 (0.00030)

T0, Body Mass at time of surgery; T1, Body Mass at time of sacrifice; ^*&*^ Body Mass at time of sacrifice was used for correlation analysis; ^*#*^Non-normally distributed data. Data are presented as mean (SD) for normally distributed data and median (IQR) for non-normally distributed data. IVD, intervertebral disc; NP, nucleus pulposus; MF, multifidus muscle; ML, longissimus muscle.

**Figure 1.**
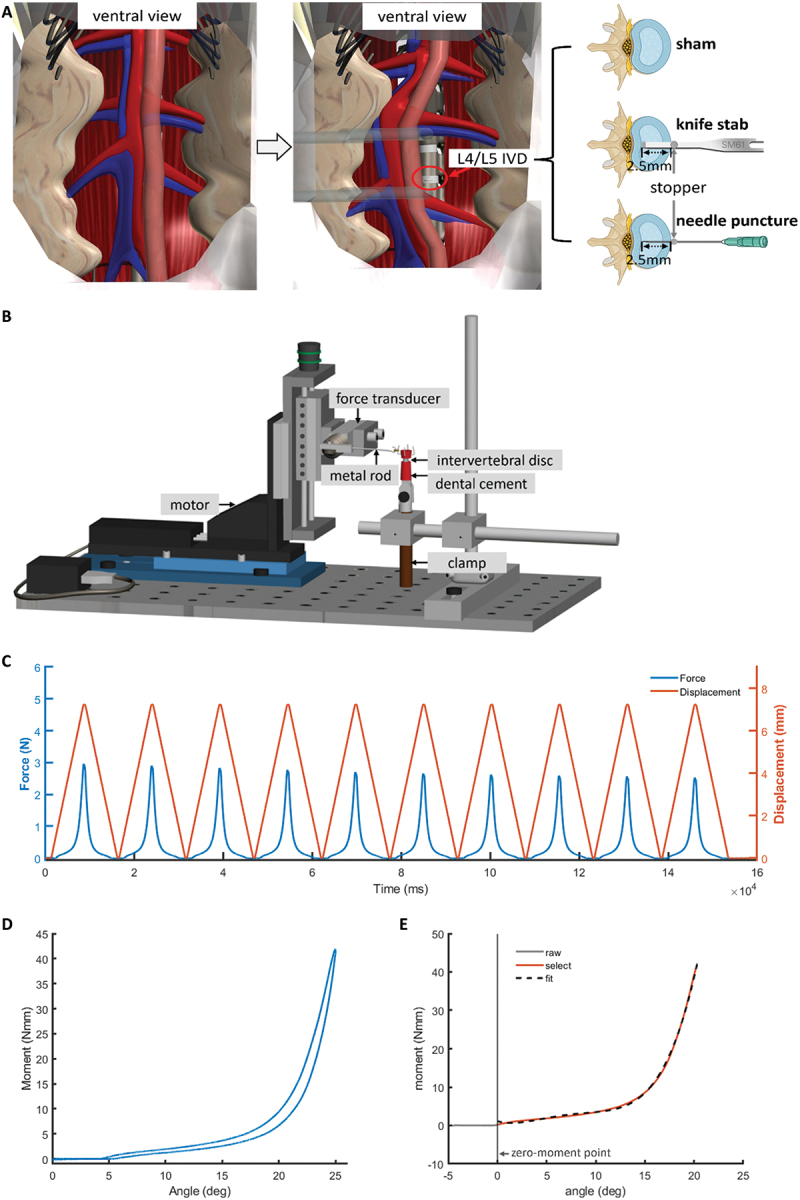
(A) Schematic of intervertebral disc injury surgical approach. Drawing was made by Guus Baan. (B) Mechanical test setup. Drawing was made by Guus Baan. (C) An example of filtered time series of force (blue) and displacement (red) signals from a flexion test. (D) Angle–moment curve of the 10th cycle from above signals. (E) Loading part of the 10th angle–moment curve with adjusted ‘zero-displacement point’. Grey, raw data; red, selected data after removing the slack angle; black dashed line, fourth-order polynomial fitted data.

In injury groups, the L4/L5 IVD was injured using a transperitoneal-ventral approach as described previously (Figure 1(A)) (Maas et al. 2018). The IVD was injured ventral-dorsally in the middle to a depth of 2.5 mm fully penetrating the NP, using a scalpel blade (Swann Morton, SM61, max. width 1.6 mm, max. thickness 0.6 mm) or a 21-gauge hypodermic needle (Becton Dickinson, outer diameter 0.8 mm) with a 360° rotation. Both the scalpel and needle had stoppers set at a depth of 2.5 mm to ensure consistent penetrating depth. Subsequently, the abdominal wound and skin were closed with absorbable sutures (5–0, Vicryl, absorbable, ETHICON). Tiny dots of tissue glue (Vetbond™ Tissue Adhesive, 3 M, Neuss, Germany) were applied between the skin sutures. The actual spine level of the injured IVD was confirmed at the time of tissue extraction. In the control group, sham surgery included all procedures necessary to access the L4/L5 IVD, but no IVD injury was induced.

### Tissue extraction

Rats in knife and needle groups were sacrificed 1 week after surgery, and those in the control group were sacrificed 2 weeks after sham surgery. 30–60 min prior to extraction of tissues, buprenorphine was administered subcutaneously. The rats were then anaesthetized using isoflurane and sacrificed with an overdose of pentobarbital (intracardial, Euthasol 20%, AST Farma, Oudewater, The Netherlands). Immediately before (under isoflurane anaesthesia) or within 2 h after sacrifice, left and right multifidus (MF) fascicles originating from L3-L4 vertebrae, as well as the medial longissimus muscles (ML) between L1-S3 were excised. The mass of the extracted muscles was measured, averaged across sides, and normalized to body mass. L4-L5 spinal segments (vertebra-IVD-vertebra) were collected and wrapped in saline-soaked gauze after removing soft tissues (muscles, supraspinous and interspinous ligaments), then stored at −20°C.

### Mechanical testing

As described previously (Xiao et al. 2023), specimens were thawed at room temperature 1 day before mechanical testing and potted in dental cement (DuraLay, Reliance Dental Manufacturing, LLC, United States), and then, wrapped in phosphate-buffered saline (PBS)-soaked gauze and stored at +4°C in sealed double plastic bags. On the day of testing, the specimens were placed at room temperature in sealed bags for 1–4 h before testing.

Non-destructive displacement-controlled bending tests of the specimens were performed in a fixed sequence: flexion, left bending, and right bending (Figure 1(B)). Maximum bending angles were 25° for flexion and 15° for lateral bending; these angles were selected based on pilot experiments and validated through in vitro mechanical testing. Passive range of motion was first measured in intact lumbar spinal segments to identify physiological limits. Mechanical responses were then evaluated via displacement-controlled tests across different angles to confirm non-destructiveness. The selected angles were further validated in our previous in vitro study assessing the acute effects of IVD injury on mechanical properties (Xiao et al. 2023). The motor moved at a constant velocity of 1 mm/s, with a maximum acceleration/deceleration of 4.5 mm/s^2^. Custom LabVIEW software (LabVIEW 13.0, National Instruments, Austin, TX) controlled motor movement and collected displacement and force signals. For each direction, 10 cycles of testing followed a 10mN preload and 10 cycles of preconditioning. In between tests, the IVD was kept moist by wrapping the IVD with PBS-soaked gauze and spraying it with PBS every 2–3 min.

### Histology

Following mechanical testing, the IVDs were isolated from the spinal segments for histology analysis, which was used to confirm the presence of disc degeneration following injury. They were frozen in liquid nitrogen and stored at −80°C. IVDs were sectioned transversally at 12 µm thickness in a cryostat at −20°C (Maas et al. 2018). Three slides from the middle portion of each IVD were stained with Picrosirius Red (PR). The slides were first dried using dry air for approximately 10 min, then fixed in Bouin’s solution for 10 min and rinsed in demineralized water. Sections were stained with 0.1% PR in saturated aqueous picric acid for 30 min, followed by sequential rinses in demineralized water, 0.01 M HCl, absolute ethanol I and II, and two xylene baths (with no less than 10 min in the second xylene rinse). Slides were then mounted with Entellan and enclosed with a cover sheet.

Images were captured under bright-field microscopy (Olympus VS200) at 20x magnification. For each sample, the IVD and NP areas were manually measured from three sections using ImageJ (National Institutes of Health, United States) by an assessor (WN) blinded to the intervention (Issy et al. 2013; Maas et al. 2018; Tavana et al. 2024). The values of the three sections were averaged, and the relative NP area was calculated as the ratio of NP to IVD area.

### Data analysis

Force and displacement signals were sampled at 100 Hz and second-order, 5 Hz, low-pass, zero-lag Butterworth filtered (Figure 1C). Angle–moment data of the 10th cycle were used for data analysis (Figure 1D), hysteresis was calculated as the area between loading and unloading curves. Subsequently, the zero-moment point was identified by removing the slack angle after preconditioning and a fourth-order polynomial was fitted to the loading part of the angle–moment curve (Figure 1E) (Xiao et al. 2023). Per direction, the lowest maximum angle across all specimens (19° for flexion, 9° for left and right bending after adjustment for zero-moment point) was used to calculate peak moment from the fitted angle–moment curve and peak stiffness from the analytical derivative of the fitted curve. All mechanical outcomes were normalized to body mass, as this was correlated to most of these outcomes (Table 2). Parameter calculations were conducted in random order by an assessor blinded to intervention.

**Table 2.** Correlation between body mass, IVD histology and IVD mechanical properties.

		Body mass (T1)	IVD height	IVD area	NP area	Relative NP area
Peak Stiffness – Flexion	Pearson’s *r*	0.541	0.018	−0.028	0.07	0.05
	df	29	28	24	24	24
	*p*-value	**0.002**	0.926	0.891	0.733	0.809
Peak Stiffness – Left bending	Pearson’s *r*	0.446	−0.232	0.004	−0.067	−0.076
	df	29	28	24	24	24
	*p*-value	**0.012**	0.216	0.986	0.744	0.712
Peak Stiffness – Right bending	Pearson’s r	0.028	−0.138	−0.198	−0.135	−0.059
	df	29	28	24	24	24
	*p*-value	0.879	0.466	0.331	0.512	0.775
Peak Moment – Flexion	Pearson’s *r*	0.546	0.008	0.093	0.072	0.012
	df	29	28	24	24	24
	*p*-value	**0.001**	0.965	0.653	0.728	0.953
Peak Moment – Left bending	Pearson’s *r*	0.446	−0.276	0.156	−0.077	−0.14
	df	29	28	24	24	24
	*p*-value	**0.012**	0.14	0.448	0.709	0.496
Peak Moment – Right bending	Pearson’s *r*	0.039	−0.155	−0.079	−0.153	−0.127
	df	29	28	24	24	24
	*p*-value	0.834	0.415	0.702	0.455	0.537
Hysteresis – Flexion	Pearson’s *r*	0.604	−0.131	−0.094	0.035	0.007
	df	29	28	24	24	24
	*p*-value	** < .001**	0.489	0.646	0.864	0.972
Hysteresis – Left bending	Pearson’s *r*	0.308	−0.258	−0.332	−0.241	−0.086
	df	29	28	24	24	24
	*p*-value	0.092	0.168	0.098	0.236	0.678
Hysteresis – Right bending	Pearson’s *r*	0.261	−0.151	−0.26	0.026	0.105
	df	29	28	24	24	24
	*p*-value	0.157	0.424	0.199	0.899	0.608

T1, Body Mass at time of sacrifice; df, degree of freedom.

### Statistical analysis

MATLAB R2021a (MathWorks, Inc., Natick, MA, United States) was used for statistical analysis. Normality was tested with the Shapiro–Wilk test; homogeneity of variance was tested with Levene’s test (Appendix A). Pearson correlation coefficients were calculated to determine associations between body mass, IVD height, IVD area, NP area, relative NP area, and IVD mechanics. The fits to the loading parts of the angle–moment curves of the 10th testing cycle of all IVDs were compared between groups using the MATLAB-based spm1d-package (SPM) (Pataky 2010). According to data distribution, parametric or non-parametric tests ‘*ex1d_*anova*1’*were used. Differences were considered significant if any SPM values exceeded the critical threshold. Depending on the data distribution, parametric (one-way ANOVA with Tukey adjusted pairwise comparisons) or non-parametric (Kruskal–Wallis with Mann–Whitney *U* test pairwise comparisons) tests were used, to evaluate differences between groups for IVD structural and mechanical properties, and muscle mass. For all comparisons, alpha was set at 0.05. Data are presented as mean (SD) or median (IQR).

## Results

No pain-related behaviors (e.g. increased grooming, ‘wet-dog shakes’) or other signs of discomfort (grimacing, reduced activity, reduced responsiveness) following IVD injury were observed in any rat throughout the experimental period. During the recovery period, several rats had chewed the skin incisions and sutures open; local wounds were treated and re-sutured immediately after observation. During tissue extraction, we found injuries in the needle group at L3/L4 in two rats and at L5/L6 in four rats. In these cases, the corresponding spinal segments were collected for mechanical and histological analysis. Body mass at termination was significantly lower in the needle group than in the control and knife groups (*p* = 0.015, *χ*^2^_(2)_ = 8.384) (Table 1).

### IVD injury induces IVD mechanical changes

No significant group differences were found in the angle–moment curves of flexion, but significant differences were found in left bending in the initial 6% of the bending angle (*p* = 0.040, *F* = 6.133) (Appendix B, Figure 1(B(1–2))) and in right bending in the initial 1.4% (*p* = 0.040, *F* = 5.507) (Appendix B, Figure 1(C(1-2))), where the needle group showed higher moments than the control group and/or the knife group. However, the moments in this toe-region of the angle–moment curves were very low, approaching zero. Hence, we do not consider this to be functionally relevant.

**Figure 2.**
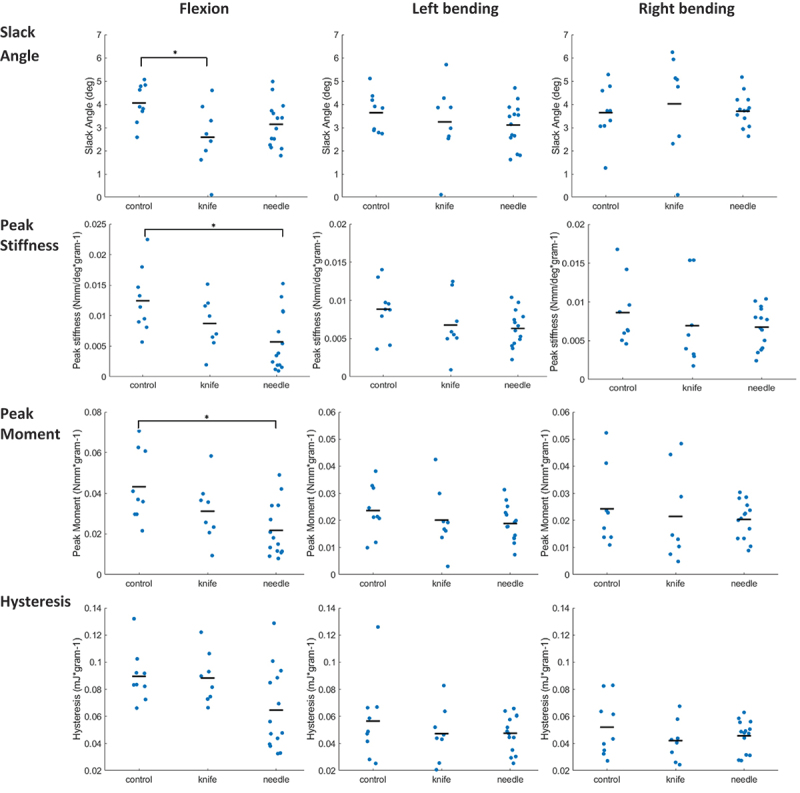
IVD mechanical properties. Dots represent individual data of each specimen, black bars represent the mean of the group. Asterisks (*) indicate a significant difference between groups.

The mechanical properties of flexion in IVD differed significantly between groups (Figure 2 and Table 3–5). (1) Slack angle was smallest in the knife group, showing a 36% (1.5°) reduction compared to the control group (*p* = 0.022, *t* = 2.850). (2) Peak stiffness differed between groups (*p* = 0.011, *F*_(2,28)_ = 5.348), with the needle group having a 54% lower stiffness than the control group (*p* = 0.008, *t* = 3.260). (3) Peak moment varied significantly among groups (*p* = 0.024, *χ*^2^_(2)_ = 8.657), with the needle group showing a 55% lower moment than the control group (*p* = 0.006, *U* = 107). (4) Hysteresis also differed between groups (*p* = 0.036, *F*_(2,28)_ = 3.735), but pairwise comparisons showed no significant differences. No significant differences were found between groups with left or right bending.

**Table 3.** Mechanical properties of IVD.

Group	Sample size	Injury at L4/L5	Slack angle (deg)			Peak stiffness (Nmm/deg·g^−1^)			Peak moment (Nmm·g^−1^)			Hysteresis (mJ·g^−1^)		
			Flexion	Left bending	Right bending^#^	Flexion	Left bending	Right bending^#^	Flexion^#^	Left bending	Right bending^#^	Flexion	Left bending^#^	Right bending
Control	9	NA	4.06 (0.83)	3.64 (0.85)	3.72 (1.52)	0.0125 (0.0053)	0.0089 (0.0035)	0.0064 (0.0036)	0.0370 (0.0311)	0.0236 (0.0094)	0.0228 (0.0098)	0.0895 (0.0192)	0.0488 (0.0248)	0.052 (0.0213)
Knife	8	8	2.58 (1.40)	3.24 (1.63)	4.91 (2.78)	0.0087 (0.0042)	0.0068 (0.0038)	0.00485 (0.0059)	0.0307 (0.0147)	0.0201 (0.0117)	0.0138 (0.0230)	0.0883 (0.0187)	0.0452 (0.0162)	0.0421 (0.0149)
Needle	14	8 (2, L3/L4; 4, L5/L6)	3.14 (0.98)	3.11 (0.95)	3.73 (0.97)	0.0057 (0.0049)	0.0063 (0.0024)	0.00717 (0.0045)	0.0165 (0.0207)	0.0189 (0.0066)	0.0215 (0.0109)	0.0646 (0.0300)	0.0481 (0.0221)	0.0456 (0.0117)

^#^Non-normally distributed data.

**Table 4.** Overview of ANOVA statistical results of IVD histology and mechanics.

Variable	F (df)	*p*-value	Effect size (η^2^)	Pairwise comparisons	t-value	p-value (Tukey adjusted)	Effect size (Cohen’s d)
IVD height	0.557 (2, 27)	0.579	0.04				
IVD area	0.643 (2, 23)	0.535	0.053				
NP area	4.969 (2, 23)	**0.016**	0.302	control – knife	0.415	0.910	0.20
				control – needle	2.934	**0.020**	1.38
				knife – needle	2.432	0.058	1.18
Slack Angle – Flexion	4.218 (2, 28)	**0.025**	0.232	control – knife	2.850	**0.022**	1.38
				control – needle	2.010	0.128	0.86
				knife – needle	−1.180	0.473	−0.52
Slack Angle – Left bending	0.614 (2, 28)	0.548	0.042				
Peak Stiffness – Flexion	5.348 (2, 28)	**0.011**	0.276	control – knife	1.590	0.268	0.77
				control – needle	3.260	**0.008**	1.40
				knife – needle	1.410	0.350	0.62
Peak Stiffness – Left bending	1.866 (2, 28)	0.173	0.118				
Peak Moment – Left bending	0.799 (2, 28)	0.460	0.054				
Hysteresis – Flexion	3.735 (2, 28)	**0.036**	0.211	control – knife	0.103	0.994	0.05
				control – needle	2.362	0.064	1.01
				knife – needle	2.164	0.095	0.96
Hysteresis – Right bending	0.888 (2, 28)	0.423	0.06				
Normalized MF mass	1.166 (2, 27)	0.327	0.079				
Normalized ML mass	5.24 (2, 26)	**0.012**	0.287	control – knife	−0.234	0.970	−0.12
				control – needle	−2.731	**0.029**	−1.26
				knife – needle	−2.580	**0.041**	−1.14

IVD, intervertebral disc; NP, nucleus pulposus; MF, multifidus muscle; ML, longissimus muscle; df, degree of freedom.

**Table 5.** Overview of Kruskal–Wallis statistical results of IVD histology and mechanics.

Variable	χ^2^ (df)	p-value	Effect size (ε^2^)	Pairwise comparisons	U statistic	p-value	Effect size (r_rb_)*
Body Mass (T1)	8.384 (2)	**0.015**	0.28	control – knife	32	0.692	0.13
				control – needle	97	**0.035**	−0.54
				knife – needle	94	**0.010**	−0.68
Relative NP area	7.497 (2)	**0.024**	0.30	control – knife	46	0.370	−0.28
				control – needle	69	**0.011**	−0.70
				knife – needle	56	0.059	−0.56
Slack Angle – Right bending	0.736 (2)	0.692	0.02				
Peak Stiffness – Right bending	1.712 (2)	0.425	0.06				
Peak Moment – Flexion	8.657 (2)	**0.013**	0.29	control – knife	52	0.139	−0.44
				control – needle	107	**0.006**	−0.70
				knife – needle	78	0.142	−0.39
Peak Moment – Right bending	0.782 (2)	0.676	0.03				
Hysteresis – Left bending	0.520 (2)	0.771	0.02				

T1, Body Mass at time of sacrifice; NP, nucleus pulposus; * r_rb_: Rank-biserial correlation; df, degree of freedom.

### IVD injury induces IVD structural changes

**Figure 3.**
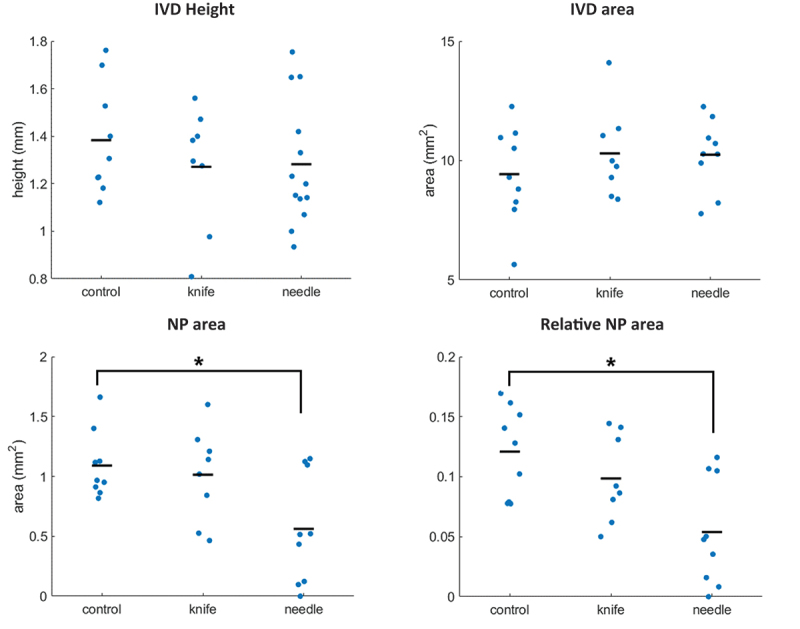
IVD structural parameters. Dots represent individual data of each specimen, black bars represent the mean of the group. Asterisks (*) indicate a significant difference between groups.

No group effects on IVD height and area were observed, but significant differences were found in absolute and relative NP areas. The needle group exhibited smaller absolute and relative NP areas than the control group (*p* = 0.020, 0.011, respectively), while no other post hoc differences were found (Figure 3 and Tables 1, 4, 5).

**Figure 4.**
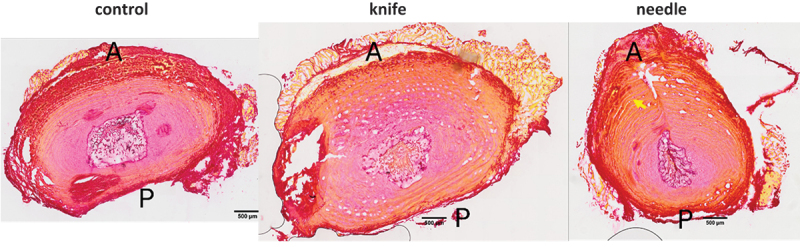
Examples of sections of the intervertebral disc stained with Picrosirius Red. Less organized annulus fibrosus (AF), less round-shaped nucleus pulposus (NP), and less clear border between AF and NP were observed in the knife and needle groups. a, anterior. p, posterior. Yellow arrow points to the needle puncture tract. Scale bar = 500 µm.

PR staining (Figure 4) showed intact and well-organized AF fiber rings, a round-shaped NP, and a clear border between AF and NP in the control group. In the knife group, the AF fiber rings were less organized, NP was less round, and the border between AF and NP at the injury site was less clear. In the needle group, the AF rings were also less organized and even ruptured, the NP lacked a round shape, the border between AF and NP was unclear, appearing mostly near the injury site, and the needle tract was visible in some specimens.

### Muscle properties

**Figure 5.**
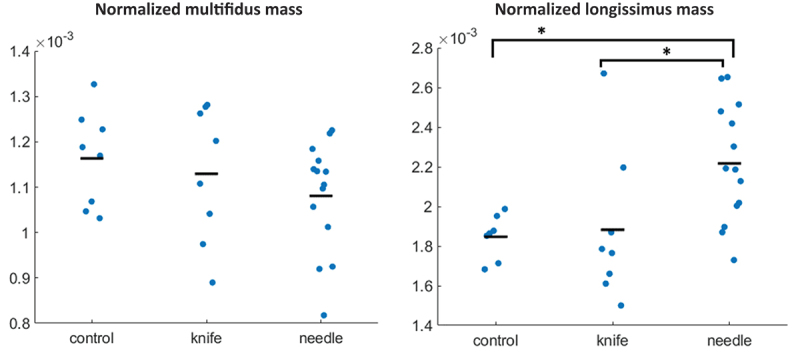
Normalized mass of multifidus (left) and longissimus (right) muscle. Dots represent individual data of each specimen, black bars represent the mean of the group. Asterisks (*) indicate a significant difference between groups.

No group effect was found in the normalized MF mass, but a significant group effect was found in the normalized ML mass (*p* = 0.012, *F*_(2,26)_ = 5.24), with the needle group having a significantly higher mass than the control (*p* = 0.029, *t* = −2.731) and knife groups (*p* = 0.041, *t* = −2.580) (Figure 5 and Table 1, 4).

## Discussion

Distinct mechanical and structural consequences were observed within 1 week after IVD injury, in particular after needle injury, supporting that this intervention provides a successful rat model of lumbar spine instability. IVD injury, using a 21 G needle, caused significant mechanical instability in flexion (decreased peak stiffness, peak moment, hysteresis), along with reduced NP size and disorganized AF. However, IVD height and area remained unchanged. No significant effects of IVD injury on left- and right-bending mechanics were observed, although structural degeneration was evident through histological analysis. With respect to muscle adaptation, there was no significant alteration in normalized MF mass, but an increased normalized ML mass was found in the needle group.

Various methods have been used to induce IVD degeneration in rodent models (Poletto et al. 2023), with degeneration severity affected by parameters, such as injury depth (Han et al. 2008), number (Ulrich et al. 2007) and size (Elliott et al. 2008; Martin et al. 2013). In this study, we compared knife stab lesions and needle punctures. Our results indicate that needle puncture changes the mechanical properties of the IVD, specifically, peak stiffness (54%) and peak moment (55%) in flexion, comparable to the 60% reduction in compressive and torsional stiffness reported after mouse tail disc injuries using a 26 G needle (Martin et al. 2013) and 30% reduction in tensile strength after rat tail disc AF injury (Xu et al. 2024). Despite the fact that the knife’s maximum thickness (equal to 43% of the average disc height in the control group) exceeded the suggested 40% threshold for inducing mechanical changes (Elliott et al. 2008), no significant effects on IVD mechanics were observed. Possibly the needle puncture created more damage to the IVD compared to the knife stab lesion. Firstly, the thickness of the knife decreases towards the tip, so even with a relatively large width, the damage in the center of the IVD might not be sufficient. In addition, the needle removed some disc tissue when rotated 360° before withdrawal. More NP tissue may have herniated through the needle tract, causing more depressurization and shrinkage of the NP (Figure 3), which could be confirmed by the smaller NP size in the needle group than in the knife group (Table 1). Previous work has shown that a single needle puncture can substantially alter the microscale strain distribution in the AF under load, which may directly impair tissue-cell mechanotransduction and contribute to early degeneration (Michalek et al. 2010).

Importantly, statistical comparisons using non-normalized data yielded the same significant group differences (*p* < 0.05), confirming that normalization did not mask differences between groups. The difference in injury effects between knife and needle may have been increased by partial healing of the AF through granulation tissue formation (Kaapa et al. 1995) or spontaneous repair (Schollum et al. 2010). For smaller defects, this repair process might close the annular tear, allowing NP re-pressurization via nuclear matrix synthesis, thereby restoring disc mechanical properties. In contrast, larger defects may require longer wound closure times and prolong the impaired disc mechanical function (Lotz 2004).

Our study quantitatively and qualitatively assessed the effects of injury on IVD morphology. In contrast to previous studies in rats, no significant injury effects on IVD height were found, with only a 7–8% height loss after injury, compared to approximately 20% height reduction after lumbar (Mosley et al. 2020) and tail (Zhu et al. 2023) IVD injuries. This discrepancy may be due to methodological differences in height assessment. In our study, IVD height was measured from 2D spinal segment images, which is less precise than MRI or X-ray imaging. Despite no changes in IVD height, other structural parameters, such as NP area and AF disorganization clearly indicated early degenerative changes. Compared to the control group, we observed a similar IVD area, but a smaller NP area in the injury groups, consistent with findings from rat (Zhang et al. 2009; Maas et al. 2018), porcine (Niinimaki et al. 2007) and rabbit (Jacobs et al. 2013) IVD injury studies. Specifically, the relative NP area in the knife and needle groups showed 30% and 63% reductions, respectively, consistent with our previous findings (Maas et al. 2018). Histological staining confirmed degenerative changes similar to those in previous studies, including disorganized AF fiber rings, fiber rupture, decreased NP area in the injured discs, as well as loss of a distinct border between AF and NP (Mosley et al. 2020; Elmounedi et al. 2022; Zhu et al. 2023). These findings indicate that our injury was sufficient to induce morphological degeneration of the IVD.

With respect to muscular responses to IVD injury, no significant effects on normalized MF mass were observed in our study. Our previous study also found no change in MF fiber type composition following the same lumbar IVD knife stab lesion in the rat (Docter et al. 2021). However, MF atrophy within 1 week following lumbar IVD injury, indicated by decreased normalized mass (Maas et al. 2018) and cross-sectional area (Hodges et al. 2006) has been reported. Interestingly, we found an increase in normalized ML mass, suggesting compensatory adaptation to maintain spinal stability. Further research is needed to explore the specific responses of the paraspinal muscles, for instance, changes in connective tissue content and mechanical properties.

One limitation of this paper is that the control group animals were 1 week older than those in the injury groups, and their body mass at the time of surgery (Table, T0) was lower in the needle group, likely due to the natural variation in body mass among animals at the time of purchase. The 2-week endpoint of the control animals was chosen to align the midpoint of the knife group follow-up intervals (1, 2, and 3 weeks). However, all rats were skeletally mature, and previous studies observed no significant changes in IVD structure (Maas et al. 2018), mechanics (Martin et al. 2013) or back muscle mass (Maas et al. 2018) within 1 week. Therefore, we do not expect this to have biased the interpretation of the mechanical outcomes. Our method for assessing disc height, although practical for this study, is not as accurate as MRI (Li et al. 2019) or X-ray (Mosley et al. 2020). For the needle group, the level of injury was not L4/L5 only. As IVD area (Maas et al. 2018) and height are similar for adjacent levels (confirmed by similar means in Table 1), this is not expected to cause bias. In addition, this study focused on short-term changes post IVD injury to establish a lumbar spine instability model. For the knife group, additional groups at 2 and 3 weeks post injury were also assessed. However, no differences were observed in muscle mass, IVD morphology, or mechanics between 1-, 2-, and 3-week groups (Appendix A: Tables 3-4). Since degenerative changes in IVD and muscular adaptations may take longer to fully manifest (Maas et al. 2018), a longer follow-up period would provide more insight into the chronic effects of IVD injury. Future studies could also incorporate longitudinal micro-CT imaging to assess vertebral bone microstructural changes over time following IVD injury (Newton et al. 2020).

Lumbar IVD injury induced mechanical instability, thereby validating a preclinical model that serves primarily as a platform for future mechanistic and interventional research. In addition, the increase in normalized ML mass suggests that muscular adaptation may occur as a compensatory response soon after IVD lesion, providing insights into the neuromuscular consequences of IVD degeneration. Histological findings serve as supportive evidence of structural disruption, however, the study was not designed to address the temporal sequence between structural and mechanical changes. This study holds potential clinical relevance by improving a mechanically validated model of lumbar spine instability, in which early structural and muscular responses can be examined in relation to IVD injury.

## Supplementary Material

Supplemental Material

## References

[cit0001] Alini M et al. 2008. Are animal models useful for studying human disc disorders/degeneration? Eur Spine J. 17(1):2–19. 17632738 10.1007/s00586-007-0414-y PMC2365516

[cit0002] Chen CH et al. 2016. Time course investigation of intervertebral disc degeneration in a rat-tail puncture model. Life Sci. 156:15–20. eng. 10.1016/j.lfs.2016.05.020 27197027

[cit0003] Chen T, Cheng X, Wang J, Feng X, Zhang L. 2018. Time-course investigation of intervertebral disc degeneration induced by different sizes of needle punctures in rat tail disc. Med Sci Monit. 24:6456–6465. Eng. 10.12659/MSM.910636 30216335 PMC6151108

[cit0004] Desmoulin GT, Pradhan V, Milner TE. 2020. Mechanical aspects of intervertebral disc injury and implications on biomechanics [review]. Spine. 45(8):E457–E464. English. 10.1097/BRS.0000000000003291 31651681

[cit0005] Docter H, van den Hout JD, Noort W, van Dieën J, Maas H. 2021. No changes in muscle fibre type composition in rat multifidus muscle following lesion of the lumbar intervertebral disc. Eur J Anat. 25(4):447–454. English.

[cit0006] Elliott DM et al. 2008. The effect of relative needle diameter in puncture and sham injection animal models of degeneration [article]. Spine. 33(6):588–596. English. 10.1097/BRS.0b013e318166e0a2 18344851

[cit0007] Elmounedi N et al. 2022. Impact of needle size on the onset and the progression of disc degeneration in rats. Pain Physician. 25(6):509–517. 36122262

[cit0008] Farfan HF, Gracovetsky S. 1984. The nature of instability. Spine (Phila Pa 1976). 9(7):714–719. 10.1097/00007632-198410000-00011 6505842

[cit0009] Faul F, Erdfelder E, Lang AG, Buchner A. 2007. G*Power 3: a flexible statistical power analysis program for the social, behavioral, and biomedical sciences. Behav Res Methods. 39(2):175–191. 17695343 10.3758/bf03193146

[cit0010] Fontana G, See E, Pandit A. 2015. Current trends in biologics delivery to restore intervertebral disc anabolism. Adv Drug Deliver Rev. 84:146–158. English. 10.1016/j.addr.2014.08.008 25174310

[cit0011] Fujii K et al. 2019. Discogenic back pain: literature review of definition, diagnosis, and treatment. JBMR Plus. 3(5):e10180. 10.1002/jbm4.10180 31131347 PMC6524679

[cit0012] Fujiwara A et al. 2000a. The effect of disc degeneration and facet joint osteoarthritis on the segmental flexibility of the lumbar spine. Spine (Phila Pa 1976). 25(23):3036–3044. 10.1097/00007632-200012010-00011 11145815

[cit0013] Fujiwara A et al. 2000b. The relationship between disc degeneration, facet joint osteoarthritis, and stability of the degenerative lumbar spine. J Spinal Disord. 13(5):444–450. English. 10.1097/00002517-200010000-00013 11052356

[cit0014] Galbusera F et al. 2014. Ageing and degenerative changes of the intervertebral disc and their impact on spinal flexibility. Eur Spine J. 23 Suppl 3:S324–332. 10.1007/s00586-014-3203-4 24482074

[cit0015] Glaeser JD et al. 2020. Optimization of a rat lumbar IVD degeneration model for low back pain. JOR Spine. 3(2):e1092. 10.1002/jsp2.1092 32613167 PMC7323460

[cit0016] Han B et al. 2008. A simple disc degeneration model induced by percutaneous needle puncture in the rat tail. Spine. 33(18):1925–1934. 10.1097/BRS.0b013e31817c64a9 18708924

[cit0017] Hartvigsen J et al. 2018. What low back pain is and why we need to pay attention. Lancet. 391(10137):2356–2367. Eng. 29573870 10.1016/S0140-6736(18)30480-X

[cit0018] Hodges P, Holm AK, Hansson T, Holm S. 2006. Rapid atrophy of the lumbar multifidus follows experimental disc or nerve root injury. Spine (Phila Pa 1976). 31(25):2926–2933. 10.1097/01.brs.0000248453.51165.0b 17139223

[cit0019] Iatridis JC, Nicoll SB, Michalek AJ, Walter BA, Gupta MS. 2013. Role of biomechanics in intervertebral disc degeneration and regenerative therapies: what needs repairing in the disc and what are promising biomaterials for its repair? Spine J. 13(3):243–262. 10.1016/j.spinee.2012.12.002 23369494 PMC3612376

[cit0020] Issy AC et al. 2013. Experimental model of intervertebral disc degeneration by needle puncture in Wistar rats. Braz J Med and Biol Res = Revista brasileira de pesquisas medicas e biologicas. 46(3):235–244. Eng. 10.1590/1414-431X20122429 23532265 PMC3854370

[cit0021] Jacobs L et al. 2013. Glucosamine supplementation demonstrates a negative effect on intervertebral disc matrix in an animal model of disc degeneration. Spine. 38(12):984–990. 10.1097/BRS.0b013e318286b31e 23324939 PMC3672267

[cit0022] Jin L, Balian G, Xdj L. 2018. Animal models for disc degeneration-an update. Histol Histopathol. 33(6):543–554. English. 28580566 10.14670/HH-11-910 PMC5975243

[cit0023] Kaapa E et al. 1995. Collagen synthesis and types I, III, IV, and VI collagens in an animal model of disc degeneration. Spine (Phila Pa 1976). 20(1):59–66; discussion 66-57. 10.1097/00007632-199501000-00011 7709281

[cit0024] Kim JS et al. 2011. The rat intervertebral disk degeneration pain model: relationships between biological and structural alterations and pain. Arthritis Res Ther. 13(5):R165. eng. 10.1186/ar3485 21996269 PMC3308099

[cit0025] Knutsson F. 1944. The instability associated with disk degeneration in the lumbar spine. Acta Radiol. 25(5–6):593–609. English. 10.3109/00016924409136488

[cit0026] Kong MH et al. 2009. Kinetic magnetic resonance imaging analysis of abnormal segmental motion of the functional Spine unit. J Neurosurg Spine. 10(4):357–365. 10.3171/2008.12.SPINE08321 19441995

[cit0027] Korecki CL, Costi JJ, Iatridis JC. 2008. Needle puncture injury affects intervertebral disc mechanics and biology in an organ culture model. Spine (Phila Pa 1976). 33(3):235–241. eng. 10.1097/BRS.0b013e3181624504 18303454 PMC2587060

[cit0028] Lai A et al. 2016. Annular puncture with tumor necrosis factor-alpha injection enhances painful behavior with disc degeneration in vivo. Spine J. 16(3):420–431. eng. 10.1016/j.spinee.2015.11.019 26610672 PMC4913353

[cit0029] Li L et al. 2019. Diffusion kurtosis imaging provides quantitative assessment of the microstructure changes of disc degeneration: an in vivo experimental study. Eur Spine J. 28(5):1005–1013. 10.1007/s00586-019-05924-3 30778770

[cit0030] Lotz JC. 2004. Animal models of intervertebral disc degeneration: lessons learned. Spine (phila Pa 1976). 29(23):2742–2750. eng. 10.1097/01.brs.0000146498.04628.f9 15564923

[cit0031] Maas H, Noort W, Hodges PW, van Dieen J. 2018. Effects of intervertebral disc lesion and multifidus muscle resection on the structure of the lumbar intervertebral discs and paraspinal musculature of the rat. J Biomech. 70:228–234. 10.1016/j.jbiomech.2018.01.004 29395230

[cit0032] Martin JT et al. 2013. Needle puncture injury causes acute and long-term mechanical deficiency in a mouse model of intervertebral disc degeneration. J Orthop Res. 31(8):1276–1282. 10.1002/jor.22355 23553925 PMC6684036

[cit0033] Michalek AJ, Buckley MR, Bonassar LJ, Cohen I, Iatridis JC. 2010. The effects of needle puncture injury on microscale shear strain in the intervertebral disc annulus fibrosus. Spine J. 10(12):1098–1105. eng. 10.1016/j.spinee.2010.09.015 20971041 PMC2991597

[cit0034] Mosley GE et al. 2019. Sex differences in rat intervertebral disc structure and function following annular puncture injury. Spine. 44(18):1257–1269. 10.1097/BRS.0000000000003055 30973506 PMC6722021

[cit0035] Mosley GE et al. 2020. Males and females exhibit distinct relationships between intervertebral disc degeneration and pain in a rat model. Sci Rep. 10(1):15120. 10.1038/s41598-020-72081-9 32934258 PMC7492468

[cit0036] Newton MD et al. 2020. Longitudinal characterization of intervertebral disc remodeling following acute annular injury in a rat model of degenerative disc disease. Connect Tissue Res. 61(6):568–576. 10.1080/03008207.2019.1635589 31232119

[cit0037] Niinimaki J et al. 2007. Quantitative magnetic resonance imaging of experimentally injured porcine intervertebral disc. Acta Radiol. 48(6):643–649. 10.1080/02841850701326933 17611872

[cit0038] Panjabi MM. 1992. The stabilizing system of the spine. Part I. Function, dysfunction, adaptation, and enhancement. J Spinal Disord. 5(4):383–389; discussion 397. doi:10.1097/00002517-199212000-00001 1490034

[cit0039] Panjabi MM. 2003. Clinical spinal instability and low back pain. J Electromyogr Kinesiol. 13(4):371–379. 10.1016/S1050-6411(03)00044-0 12832167

[cit0040] Passias PG et al. 2011. Segmental lumbar rotation in patients with discogenic low back pain during functional weight-bearing activities. J Bone Joint Surg Am. 93(1):29–37. 10.2106/JBJS.I.01348 21209266 PMC3004094

[cit0041] Pataky TC. 2010. Generalized n-dimensional biomechanical field analysis using statistical parametric mapping. J Biomech. 43(10):1976–1982. 20434726 10.1016/j.jbiomech.2010.03.008

[cit0042] Poletto DL, Crowley JD, Tanglay O, Walsh WR, Pelletier MH. 2023. Preclinical in vivo animal models of intervertebral disc degeneration. Part 1: a systematic review. JOR Spine. 6(1):e1234. 10.1002/jsp2.1234 36994459 PMC10041387

[cit0043] Rousseau MA et al. 2007. Stab incision for inducing intervertebral disc degeneration in the rat. Spine (Phila Pa 1976). 32(1):17–24. eng. doi:10.1097/01.brs.0000251013.07656.45 17202887

[cit0044] Schollum ML, Appleyard RC, Little CB, Melrose J. 2010. A detailed microscopic examination of alterations in normal anular structure induced by mechanical destabilization in an ovine model of disc degeneration. Spine (Phila Pa 1976). 35(22):1965–1973. eng. doi:10.1097/BRS.0b013e3181e0f085 20959777

[cit0045] Shi C et al. 2018. Animal models for studying the etiology and treatment of low back pain. J Orthop Res. 36(5):1305–1312. eng.doi:10.1002/jor.23741 28921656 PMC6287742

[cit0046] Stokes IA, Gardner-Morse M. 2003. Spinal stiffness increases with axial load: another stabilizing consequence of muscle action. J Electromyogr Kinesiol. 13(4):397–402. 10.1016/S1050-6411(03)00046-4 12832169

[cit0047] Swanson BT, Creighton D. 2020. The degenerative lumbar disc: not a disease, but still an important consideration for OMPT practice: a review of the history and science of discogenic instability. J Man Manip Ther. 28(4):191–200. 10.1080/10669817.2020.1758520 32364465 PMC8550621

[cit0048] Tanaka N et al. 2001. The relationship between disc degeneration and flexibility of the lumbar spine. Spine J. 1(1):47–56. 10.1016/S1529-9430(01)00006-7 14588368

[cit0049] Tavana S, Shek C, Rahman T, Baxan N, Newell N. 2024. The influence of geometry on intervertebral disc stiffness. J Biomech. 163:111915. 10.1016/j.jbiomech.2023.111915 38233311

[cit0050] Ulrich JA, Liebenberg EC, Thuillier DU, Lotz JC. 2007. Issls prize winner: repeated disc injury causes persistent inflammation. Spine (Phila Pa 1976). 32(25):2812–2819. 10.1097/BRS.0b013e31815b9850 18246002

[cit0051] van Dieen JH, Reeves NP, Kawchuk G, van Dillen LR, Hodges PW. 2019. Motor control changes in low back pain: divergence in presentations and mechanisms. J Orthop Sports Phys Ther. 49(6):370–379. 10.2519/jospt.2019.7917 29895230 PMC7393576

[cit0052] van Dieen JH, Selen LP, Cholewicki J. 2003. Trunk muscle activation in low-back pain patients, an analysis of the literature. J Electromyogr Kinesiol. 13(4):333–351. 10.1016/S1050-6411(03)00041-5 12832164

[cit0053] Wang DL et al. 2022. Ex vivo biomechanical evaluation of acute lumbar endplate injury and comparison to annulus fibrosus injury in a rat model [article]. J Mech Behav Biomed Mater. 131:9. English. doi:10.1016/j.jmbbm.2022.105234 PMC9210935 35462160

[cit0054] Xiao F, van Dieën JH, Han J, Maas H. 2023. Stab lesion of the L4/L5 intervertebral disc in the rat causes acute changes in disc bending mechanics. J Biomech. 161:111830. 10.1016/j.jbiomech.2023.111830 37821333

[cit0055] Xu H et al. 2024. A novel rat model of annulus fibrosus injury for intervertebral disc degeneration. Spine J. 24(2):373–386. 10.1016/j.spinee.2023.09.012 37797841

[cit0056] Yerramalli CS et al. 2007. The effect of nucleus pulposus crosslinking and glycosaminoglycan degradation on disc mechanical function. Biomech Model Mechanobiol. 6(1–2):13–20. eng. doi:10.1007/s10237-006-0043-0 16715318

[cit0057] Zhang H, La Marca F, Hollister SJ, Goldstein SA, Lin C-Y. 2009. Developing consistently reproducible intervertebral disc degeneration at rat caudal spine by using needle puncture. J Neurosurg Spine. 10(6):522–530. 10.3171/2009.2.SPINE08925 19558284

[cit0058] Zhu P et al. 2023. A minimally invasive annulus fibrosus needle puncture model of intervertebral disc degeneration in rats. World Neurosurg. 169:e1–e8. 10.1016/j.wneu.2022.09.062 36283650

