# Supplementary material for: Mechanical instability of the lumbar spine following intervertebral disc injury: a comparison of two injury methods
Source: Int Biomech. 2025 Oct 30;12(1):67–80. doi: 10.1080/23335432.2025.2581469 (PMC12576908; doi:10.1080/23335432.2025.2581469)
Supplement: Supplemental Material [file TBBE_A_2581469_SM4753.zip › Supplementary Material/Appendix A_R1.docx]

**Appendix A**

| **Table 1. Normality Test (Shapiro-Wilk)** | | |
| --- | --- | --- |
|  | **W** | **p** |
| Body Mass (T1) | 0.903 | 0.009 |
| IVD Height | 0.972 | 0.585 |
| IVD area | 0.995 | 1.000 |
| NP area | 0.935 | 0.100 |
| Relative NP area | 0.909 | 0.025 |
| Slack Angle – Flexion | 0.987 | 0.959 |
| Slack angle – Left bending | 0.968 | 0.476 |
| Slack angle – Right bending | 0.952 | 0.181 |
| Peak Stiffness – Flexion | 0.935 | 0.059 |
| Peak Stiffness – Left bending | 0.971 | 0.549 |
| Peak Stiffness – Right bending | 0.904 | 0.009 |
| Peak Moment – Flexion | 0.922 | 0.027 |
| Peak Moment – Left bending | 0.977 | 0.736 |
| Peak Moment – Right bending | 0.917 | 0.020 |
| Hysteresis – Flexion | 0.932 | 0.051 |
| Hysteresis – Left bending | 0.913 | 0.015 |
| Hysteresis – Right bending | 0.954 | 0.197 |
| Normalized mass MF | 0.937 | 0.076 |
| Normalized mass LG | 0.964 | 0.421 |
| *T1, Body Mass at time of termination; IVD, intervertebral disc; NP, nucleus pulposus; MF, multifidus muscle; LG, longissimus muscle* | | |

| **Table 2. Homogeneity of Variances Test (Levene's)** | | | | |
| --- | --- | --- | --- | --- |
|  | **F** | **df1** | **df2** | **p** |
| Body Mass (T1) | 2.218 | 2 | 28 | 0.128 |
| IVD Height | 0.121 | 2 | 27 | 0.886 |
| IVD area | 0.547 | 2 | 23 | 0.586 |
| NP area | 1.433 | 2 | 23 | 0.259 |
| Relative NP area | 0.305 | 2 | 23 | 0.740 |
| Slack Angle – Flexion | 0.853 | 2 | 28 | 0.437 |
| Slack angle – Left bending | 1.340 | 2 | 28 | 0.278 |
| Slack angle – Right bending | 7.610 | 2 | 28 | 0.002 |
| Peak Stiffness – Flexion | 0.195 | 2 | 28 | 0.824 |
| Peak Stiffness – Left bending | 0.606 | 2 | 28 | 0.552 |
| Peak Stiffness – Right bending | 2.283 | 2 | 28 | 0.121 |
| Peak Moment – Flexion | 0.544 | 2 | 28 | 0.587 |
| Peak Moment – Left bending | 0.819 | 2 | 28 | 0.451 |
| Peak Moment – Right bending | 4.594 | 2 | 28 | 0.019 |
| Hysteresis – Flexion | 3.074 | 2 | 28 | 0.062 |
| Hysteresis – Left bending | 1.301 | 2 | 28 | 0.288 |
| Hysteresis – Right bending | 3.688 | 2 | 28 | 0.038 |
| Normalized mass MF | 0.872 | 2 | 27 | 0.430 |
| Normalized mass LG | 2.854 | 2 | 26 | 0.076 |
| *T1, Body Mass at time of termination; IVD, intervertebral disc; NP, nucleus pulposus; MF, multifidus muscle; LG, longissimus muscle; df, degree of freedom* | | | | |

| **Table 3.** Body mass, muscle mass, IVD histologic and mechanical properties after 2 and 3 weeks post knife stab | | | |
| --- | --- | --- | --- |
|  |  | 2w | 3w |
|  | sample size | 7 | 7 |
|  | BW (T1, gram) | 413(32) | 412(70) |
|  | IVD Height (mm) | 1.35(0.319) | 1.23(0.179) |
|  | IVD area (mm^2^) | 9.75(1.52) | 10.90(2.74) |
|  | NP area (mm^2^) | 0.881(0.176) | 0.915(0.304) |
|  | Relative NP area | 0.093(0.022) | 0.087(0.033) |
|  | Normalized MF mass | 0.00112(0.00007) | 0.00124(0.00008) |
|  | Normalized ML mass | 0.00170(0.00036) | 0.00175(0.00039) |
| Slack Angle | Flexion | 3.05(0.96) | 3.46(0.85) |
| (deg) | Left bending | 3.12(0.81) | 3.57(0.82) |
|  | Right bending | 3.67(0.89) | 3.62(0.59) |
| Peak Stiffness | Flexion | 0.0081(0.0054) | 0.0076(0.0052) |
| (Nmm/deg*gram^-1^) | Left bending | 0.0087(0.0059) | 0.0085(0.0053) |
|  | Right bending | 0.0109(0.0056) | 0.0068(0.0029) |
| Peak Moment | Flexion | 0.0275(0.0169) | 0.0266(0.0177) |
| (Nmm*gram^-1^) | Left bending | 0.0250(0.0162) | 0.0254(0.0166) |
|  | Right bending | 0.0268(0.0133) | 0.0198(0.0093) |
| Hysteresis | Flexion | 0.0898(0.0190) | 0.0861(0.0290) |
| (mJ*gram^-1^) | Left bending | 0.0534(0.0167) | 0.0553(0.0227) |
|  | Right bending | 0.0567(0.0213) | 0.0440(0.0099) |
| *T1, Body Mass at time of termination; IVD, intervertebral disc; NP, nucleus pulposus; MF, multifidus muscle; LG, longissimus muscle* | | | |

| **Table 4**. Overview of statistical results of IVD histology and mechanics after 1, 2, and 3 weeks post knife stab | | | |
| --- | --- | --- | --- |
| **ANOVA** | **F (df)** | **P-value** | **Effect Size** (η²) |
| Body Mass (T1) | 0.039(2,19) | 0.962 | 0.004 |
| IVD Height | 0.420(2,19) | 0.663 | 0.042 |
| IVD area | 0.569(2,19) | 0.576 | 0.056 |
| NP area (mm2) | 0.386(2,19) | 0.685 | 0.039 |
| Relative NP area | 0.269(2,19) | 0.767 | 0.028 |
| Normalized ML mass | 0.470(2,19) | 0.632 | 0.047 |
| slack angle - Flexion | 1.170(2,19) | 0.332 | 0.110 |
| slack angle - Right bending | 0.181(2,19) | 0.836 | 0.019 |
| Peak Stiffness - Right bending | 1.680(2,19) | 0.214 | 0.150 |
| Hysteresis - Flexion | 0.048(2,19) | 0.953 | 0.005 |
| Hysteresis - Left bending | 0.340(2,19) | 0.716 | 0.035 |
| Hysteresis - Right bending | 1.790(2,19) | 0.195 | 0.158 |
| **Kruskal-Wallis** | **χ² (df)** | **P-value** | **Effect Size** (ε²) |
| Normalized MF mass | 5.144(2) | 0.076 | 0.25 |
| slack angle - Right bending | 1.235(2) | 0.539 | 0.06 |
| Peak Stiffness - Flexion | 0.625(2) | 0.732 | 0.03 |
| Peak Stiffness - Left bending | 0.169(2) | 0.919 | 0.01 |
| Peak Moment - Flexion | 0.672(2) | 0.715 | 0.04 |
| Peak Moment - Left bending | 0.300(2) | 0.861 | 0.02 |
| Peak Moment - Right bending | 1.660(2) | 0.436 | 0.08 |
| *T1, Body Mass at time of sacrifice; IVD, intervertebral disc; NP, nucleus pulposus; MF, multifidus muscle; ML, longissimus muscle; df, degree of freedom* | | | |
