# Supplementary material for: Mechanical instability of the lumbar spine following intervertebral disc injury: a comparison of two injury methods
Source: Int Biomech. 2025 Oct 30;12(1):67–80. doi: 10.1080/23335432.2025.2581469 (PMC12576908; doi:10.1080/23335432.2025.2581469)
Supplement: Supplemental Material [file TBBE_A_2581469_SM4753.zip › Supplementary Material/Appendix B.docx]

**Appendix B**

| **A** |  |
| --- | --- |
| **B**.1 |  |
| **B**.2 | 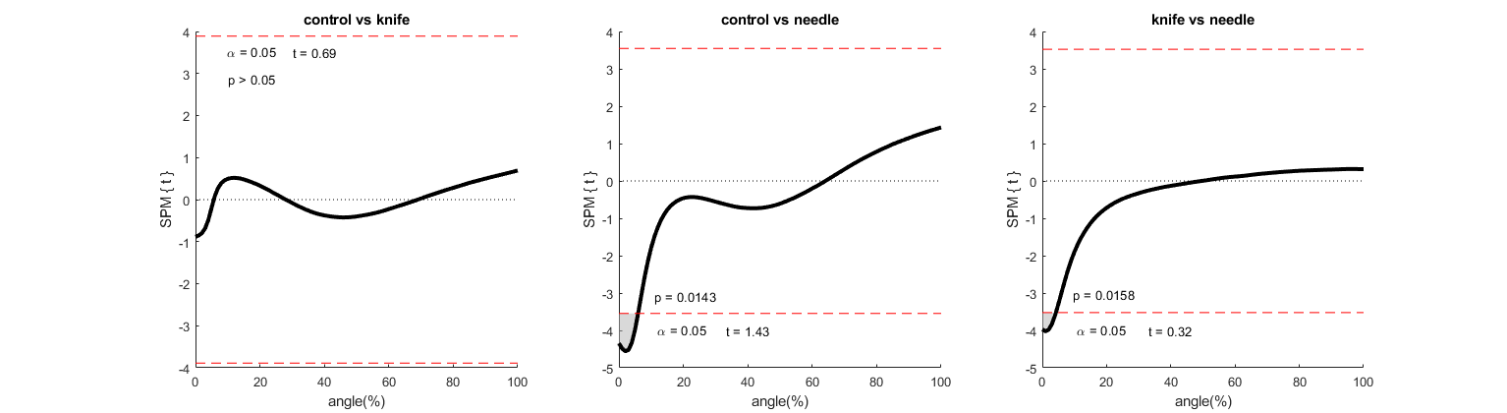 |
| **C.**1 |  |
| **C.**2 | 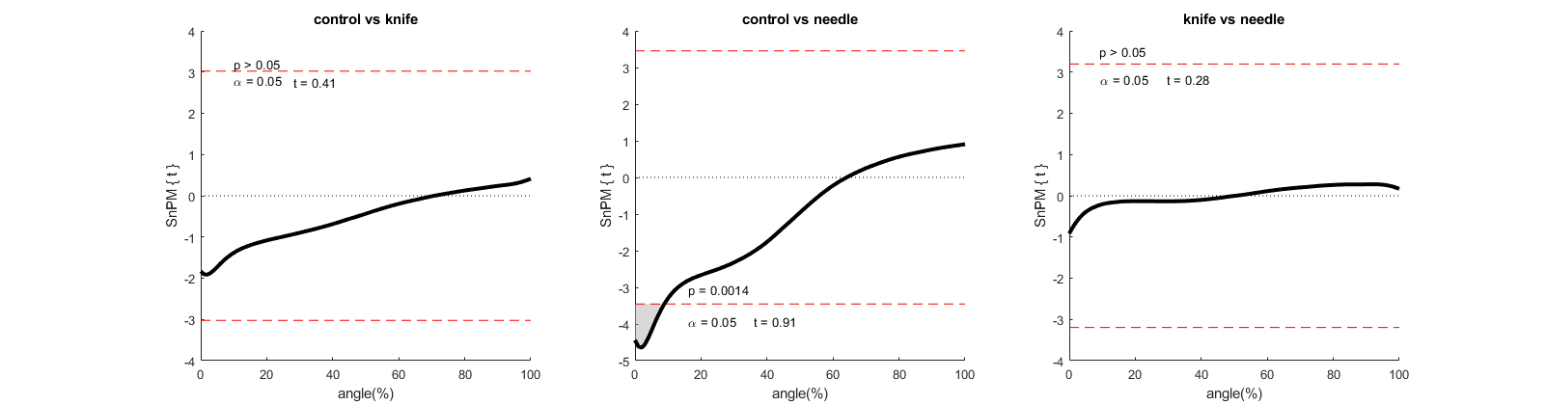 |
| **Fig.1**. Comparison of angle-moment curves in **(A)** flexion, **(B**.1**)** left bending and **(C.**1**)** right bending. Moments are plotted as a function of normalized bending angle and presented as mean with 95 % confidence interval (shaded area). Pairwise comparison of different groups in left bending (**B**.2) and right bending (**C**.2). The critical threshold was set as α=0.05 (red dashed line). Gary zone indicated region with statistically significant difference. | |
